# Domain-aligned self-supervision: CT-specialized DINOv3 enhances PET/CT tumor segmentation

**DOI:** 10.1088/1361-6560/aeb29f

**Published:** 2026-10-23

**Authors:** Yu-Nong Scarlett Lin, Shansong Wang, Yuheng Li, Mojtaba Safari, Qiang Li, Zach Eidex, Yizhou Wu, Ryan Sanford, Kevin T Chen, Xiaofeng Yang

**Affiliations:** 1 Department of Biomedical Engineering, Georgia Institute of Technology, Atlanta, GA, United States of America; 2 Department of Radiation and Cellular Oncology, The University of Chicago, Chicago, IL, United States of America; 3 Department of Radiation Oncology and Winship Cancer Institute, Emory University, Atlanta, GA, United States of America; 4 Department of Computer Science, Emory University, Atlanta, GA, United States of America; 5 Department of Electrical and Computer Engineering, Georgia Institute of Technology, Atlanta, GA, United States of America; 6 School of Mechanical Engineering, Georgia Institute of Technology, Atlanta, GA, United States of America; 7 Department of Biomedical Engineering, National Taiwan University, Taipei, Taiwan; 8 Center for Advanced Computing and Imaging in Biomedicine, National Taiwan University, Taipei, Taiwan

**Keywords:** foundation model, PET/CT tumor segmentation, DINOv3, self-supervised learning, nnUNet

## Abstract

*Objective.* This study investigates how domain-aligned self-supervised pretraining influences downstream PET/CT tumor segmentation, with a focus on disentangling the roles of domain proximity and pretraining dataset scale. *Approach.* We compared nnU-Net segmentation models initialized from random weights or from DINOv3 encoders pretrained on natural images, x-ray mammography, CT, or PET, using datasets of different sizes. To disentangle the effects of pretraining domain and dataset size, CT-DINOv3 encoders were pretrained using different numbers of 2D CT slices, including subsets matched in size to the pretraining datasets from other domains. Models were evaluated on two PET/CT benchmarks, autoPET (whole-body, FDG/prostate-specific membrane antigen (PSMA)) and HECKTOR (head-and-neck primary tumors and metastatic lymph nodes), using five-fold cross-validation with frozen encoders. *Main results.* CT-pretrained models achieved the highest Dice scores for lymph nodes, FDG lesions, and PSMA lesions. For primary tumors, PET pretraining produced the highest observed Dice score, followed by CT pretraining, suggesting the potential benefit of in-domain representations when PET data are sufficient. Notably, PET pretraining exceeded size-matched CT pretraining for PSMA but remained below models pretrained on larger CT datasets for both PSMA and FDG targets. In contrast, natural-image pretraining did not outperform from-scratch training despite its much larger pretraining scale, indicating that scale alone is insufficient when domain proximity is low. Across comparisons, strong transfer reflected the combination of domain proximity and sufficient pretraining scale, especially for CT-based models with broad data availability. *Significance.* These results demonstrate that both domain proximity and dataset scale jointly determine the effectiveness of self-supervised pretraining. While higher domain alignment improves performance at comparable scale, sufficient data is required to realize this benefit. CT-based pretraining provides a favorable balance between domain relevance and data availability, leading to robust performance across tasks. This study provides practical guidance for selecting pretraining strategies in PET/CT segmentation.

## 1. Introduction

PET/CT is integral to modern clinical workflows, providing critical information for diagnosis, staging, and treatment planning for malignant tumors and metastatic disease. This hybrid imaging modality synergistically combines high-resolution anatomical information from CT with functional metabolic data from PET, enabling a comprehensive characterization of tumor biology within its anatomical context. ^18^F-fluorodeoxyglucose (FDG) is the most dominantly used PET radiotracers in oncology (Boellaard *et al* 2014), especially for head-and-neck, lung, and other metastatic lesions, due to its ability to capture fundamental alterations in glucose metabolism that are common across neoplasms. In addition, prostate-specific membrane antigen (PSMA)-targeted PET has emerged as a highly sensitive radiotracer for prostate cancer , supporting disease staging, recurrence evaluation, and selection for PSMA-targeted radiopharmaceutical therapy (Gafita *et al* 2025). Previous studies have shown PET/CT enables more accurate delineation of gross tumor volumes than anatomical imaging alone (Lardinois *et al* 2003, Guido *et al* 2009), and that incorporating PET into radiation treatment planning improves the identification of high-risk tumor subregions and can lead to better clinical outcomes (van den Bosch *et al* 2020, Ajdari *et al* 2022, Trotter *et al* 2023).

To further accelerate the adoption of automated PET/CT analysis, several public benchmarks such as the Head and Neck Tumor Segmentation (HECKTOR) 2022 Challenge and the Automated Whole-Body Lesion Segmentation Challenge (autoPET) have been established to foster the development of robust and generalizable tumor segmentation pipelines. Prior work has explored U-Net architectures that incorporate either convolutional or Transformer-based blocks (Salmanpour *et al* 2023, Srivastava *et al* 2023). Among these, the nnU-Net framework (Isensee *et al* 2020) is widely regarded as the strongest supervised baseline due to its automated configuration system and robust out-of-the-box performance across diverse biomedical segmentation tasks (Jiang *et al* 2023). However, despite task-specific adaptations such as cross-modality fusion, organ masking, or post-processing rules, segmentation performance on clinically important cases, particularly small, low-uptake lesions or complex head-and-neck tumors, remains limited. Even with optimized nnU-Net pipelines, Dice scores for challenging primary tumors frequently plateau below 0.8 (Andrearczyk *et al* 2023, Rebaud *et al* 2023), and performance is sensitive to dataset size, imaging heterogeneity, and scanner differences. These limitations reflect the intrinsic difficulty of training supervised models from scratch using only task-specific labeled datasets, which are costly to curate at scale.

Prior studies on transfer learning in medical image segmentation have shown that pretrained representations can improve downstream performance by providing more stable and transferable features (Karimi *et al* 2021). These challenges have motivated growing interest in pretrained vision foundation models to overcome data scarcity (Bommasani 2021, Nguyen *et al* 2024), improve robustness across patient cohorts, and provide more expressive representations (Zhou *et al* 2023) than those learned solely from supervised task-specific objectives. Foundation models trained at large scale, typically with self-supervised learning (SSL), can extract generalizable semantic and structural priors from millions of unlabeled images, enabling more efficient downstream fine-tuning and higher performance in low-label regimes. Several families of pretraining objectives relevant to medical imaging included masked autoencoders that reconstruct images from sparsely observed inputs, encouraging the model to capture global structural representations (He *et al* 2021). Related masked image modeling approaches, such as SimMIM (Xie *et al* 2022), use pixel-level prediction with lightweight regression heads to obtain strong visual embeddings. Contrastive frameworks such as SimCLR (Chen *et al* 2020) and non-contrastive methods such as BYOL (Grill *et al* 2020) learn invariances across augmented views, promoting semantic consistency and inter-instance discrimination. Hybrid distillation–reconstruction methods, such as the DINO series (Caron *et al* 2021, Darcet *et al* 2024, Siméoni *et al* 2025), combine contrastive invariance with prototype-guided clustering to capture both global semantics and fine-grained spatial information. These SSL paradigms differ in the visual biases they induce, which in turn affects their suitability for downstream modalities and tasks.

Several foundation models trained with these objectives on natural image datasets such as the Segment Anything Model (SAM) (Kirillov *et al* 2023) and DINOv3 have been adapted to medical imaging. SAM’s promptable design enables zero-shot or interactive segmentation across several modalities (Ahamed *et al* 2023, Ma *et al* 2024, Ren *et al* 2024, Fang *et al* 2026, Zhang *et al* 2025b). In contrast, DINOv3 offers strong self-supervised representation learning without requiring manual annotations. However, recent studies (Liu *et al* 2025, Zhang *et al* 2025a) evaluating natural-image-pretrained DINOv3 on medical benchmarks report that, while it performs competitively for x-ray and CT classification, it performs substantially worse on PET segmentation tasks. This degradation may arise primarily from the pronounced mismatch between pretraining and downstream domains: PET is a functional imaging modality characterized by focal tracer uptake patterns, low spatial resolution, and minimal texture, whereas natural images emphasize edges, surfaces, and high-frequency detail. Such modality gaps reduce the transferability of natural-image-based representations and highlight the need for domain-aligned pretraining at scale.

Previously, only a limited number of foundation models have explicitly targeted PET segmentation (Noh and Lee 2025). SegAnyPET (Zhang *et al* 2025b) trained on a private PETS-5k dataset comprising 5731 whole-body PET volumes and over 1.3 M 2D slices, represents one of the first large-scale, PET-specific foundation models. Leveraging a SAM-inspired promptable architecture, it performs universal 3D organ segmentation from PET images and can delineate both seen and unseen targets using sparse interactive points, outperforming task-specific supervised models and several general-purpose foundation models on organ-level benchmarks. Nevertheless, this approach necessitates access to exceptionally large PET datasets, which remain unattainable for most institutions due to limited data availability, proprietary scanners, and heterogeneous reconstruction protocols.

To address smaller-scale pretraining settings at approximately 1k paired scans, Oh *et al* (2025) proposed a cross-domain PET/CT framework that leverages paired multimodal inputs to learn joint representations. While effective, this dependency constrains applicability in scenarios where paired acquisitions are unavailable or where radiotracer heterogeneity disrupts consistent cross-modality alignment. MedDINOv3 (Li *et al* 2025), a recent adaptation of the DINOv3 framework to the medical domain, overcame these constraints by pretraining exclusively on millions of CT slices. Their extensive experiments demonstrate strong performance in CT-based organ and tumor segmentation, suggesting that large-scale, domain-proximal medical imaging data may provide more effective model initialization for PET/CT tasks than natural-image pretraining.

Here, we define domain proximity as the degree of similarity between the pretraining and downstream domains in terms of imaging modality characteristics, acquisition physics, and anatomical semantic structure relevant to the target task. Motivated by this definition, we hypothesize that downstream PET/CT segmentation performance may benefit from CT-based pretraining compared with natural-image pretraining, even in the absence of PET-specific pretraining data.

Motivated by these considerations, this study systematically investigates the impact of domain proximity during pretraining on downstream PET/CT segmentation performance across diverse anatomical regions and imaging contexts, while explicitly considering pretraining dataset scale as a potential confound. The primary contributions of this work are as follows:

• We perform a comprehensive evaluation comparing (i) task-specific models trained from scratch, (ii) models initialized with general-domain vision foundation models, and (iii) models initialized with domain-specific medical imaging foundation models.
• We evaluate the joint effects of imaging domain and pretraining dataset size by performing domain-specific pretraining on x-ray, CT, and PET data, including PET-0.3 M for HECKTOR, PET-0.01 M for autoPET, a matched x-ray-0.4 M versus CT-0.4 M comparison, and three ordered CT scale points for each downstream dataset.
• We perform extensive benchmarking across multiple public datasets with heterogeneous radiotracers and tumor volumes to ensure robust and clinically relevant findings.
• We provide detailed stratification by lesion size and anatomical site, offering critical insights into the performance characteristics of foundation models across clinically challenging scenarios.

## 2. Material and methods

This study employed a two-stage learning framework (figure 1) designed to systematically evaluate how domain-specific self-supervised pretraining and further domain-aligned post-training can influence downstream PET/CT tumor segmentation. In the first stage, we organized DINOv3 encoder variants with increasing levels of domain proximity relative to PET: (i) A natural-image pretrained model (DINOv3) representing low proximity (Siméoni *et al* 2025), (ii) An in-house mammography post-trained model (x-ray DINOv3) representing medium proximity), (iii) CT post-trained models representing high proximity (Li *et al* 2025), and (iv) A PET post-trained model (PET DINOv3) representing in-domain proximity. CT post-training was evaluated at multiple scales, including CT-0.01 M, CT-0.4 M, CT-1 M, and CT-3 M, to distinguish domain effects from pretraining data-scale effects. CT-0.4 M controls for the x-ray-0.4 M pretraining-size comparison, CT-1 M provides an intermediate HECKTOR scale point, and CT-0.01 M provides a size-matched autoPET comparison with PET-0.01 M. In the second stage, each DINOv3 encoder was integrated into the nnU-Net segmentation pipeline and further adapted through supervised finetuning for PET/CT lesion segmentation, while maintaining encoder weights frozen to isolate the effect of the pretrained representation.

**Figure 1.**
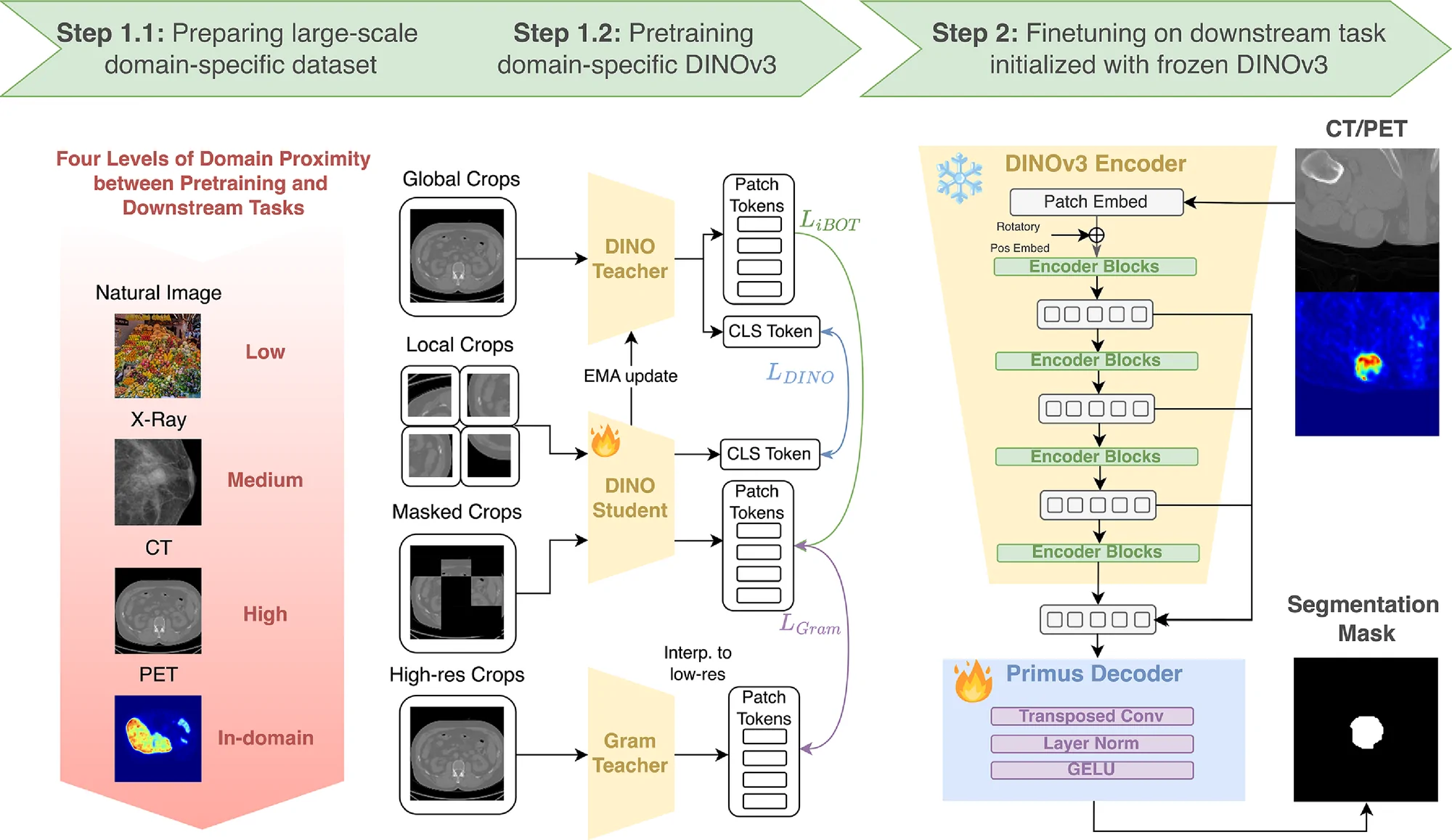
Experimental framework for evaluating domain alignment between pretraining and finetuning tasks. Stage 1 (domain-aligned pretraining): Pretraining datasets with four increasing levels of domain proximity to PET/CT are constructed, and corresponding DINOv3 encoders are initialized either from natural-image pretraining or further post-trained on medical imaging modalities to progressively increase domain alignment. CT pretraining is evaluated at both 0.4 M and 3 M samples to separate the effect of modality proximity from the effect of pretraining dataset scale. Stage 2 (downstream finetuning): The DINOv3 encoders are frozen and integrated with the Primus decoder, and the resulting models are finetuned on downstream PET/CT tumor segmentation tasks to systematically assess the impact of pretraining domain proximity on segmentation performance.

The ordering of domain proximity reflects both anatomical coverage and, more importantly, the semantic alignment of the pretraining data with the downstream PET/CT segmentation task. The highest level, in-domain PET, is defined by the fact that lesion annotations are primarily delineated based on PET tracer uptake, meaning the segmentation target is directly derived from PET signal. Thus, PET data provides the closest possible alignment. CT is considered high proximity because it offers whole-body anatomical context that closely matches the spatial coverage of PET/CT scans and supports lesion localization. However, it does not encode the functional uptake patterns that define the segmentation labels, making it inherently less aligned with the task than PET despite similar anatomical scope. Mammography is categorized as medium proximity, as it remains within the medical imaging domain but is restricted to breast-specific, organ-level imaging with limited anatomical diversity. Finally, natural images represent the lowest proximity, as their imaging principles and applications differ fundamentally from medical imaging.

Importantly, this ordering provides a framework to interpret how pretraining influences downstream performance. Methodologically, it highlights that pretraining domains with higher semantic alignment to the target task can improve feature relevance, but this effect must be considered alongside dataset scale. Accordingly, the controlled domain-proximity comparison is made primarily between x-ray-0.4 M and CT-0.4 M, while the CT-scale ablations are interpreted as scale/diversity comparisons within the same modality. Clinically, this suggests that models pretrained on anatomically and functionally relevant data may better capture lesion characteristics used in practice, potentially improving reliability across diverse tumor presentations.

Section 2.1 details the datasets used for both pretraining and finetuning. Section 2.2 outlines the self-supervised pretraining objectives and implementation details of DINOv3, while section 2.3 characterizes the downstream finetuning pipeline within frozen encoder constraints. Finally, section 2.4 presents the comparative experiments that benchmark DINOv3 models with differing domain proximity to the downstream PET/CT segmentation tasks.

### 2.1. Dataset descriptions

#### 2.1.1. Pretraining dataset

Our self-supervised pretraining leveraged three medical imaging modalities (including x-ray, CT, and PET), representing distinct points on the domain proximity spectrum relative to PET-avid tumor segmentation on PET/CT. To ensure fair comparisons, subjects used for pretraining were entirely excluded from those used for downstream evaluation.

To represent the medium domain-proximity level, the EMDB dataset (Jeong *et al* 2023) comprised 0.4 million breast-view x-ray images from 44 000 unique patients.

For the high domain proximity level, the CT pretraining data were sourced from multiple public datasets and organized into three preprocessed subsets supplied by the original MedDINOv3 authors (Li *et al* 2025): (i) AbdomenAtlas 3.0, containing 2923 917 slices (75.58%); (ii) TotalSegmentator, containing 306 022 slices (7.91%); and (iii) The universal collection, containing 638 894 slices (16.51%). The universal collection comprised the smaller public cohorts, including BTCV, Pancreas-CT, CHAOS, LiTS, KiTS, WORD, AbdomenCT-1 K, AMOS22, the CT tasks from the Medical Segmentation Decathlon, and CT-ORG. Together, these subsets contained 3868 833 slices and constituted the full CT pretraining collection, hereafter denoted CT-3 M.

To evaluate the effect of CT pretraining scale, we constructed nested CT-0.01 M, CT-0.4 M, and CT-1 M subsets by proportional random downsampling within each of the three preprocessing subsets. CT-0.01 M contained 7558 AbdomenAtlas 3.0 slices, 791 TotalSegmentator slices, and 1651 universal slices, yielding exactly 10 000 slices. CT-0.4 M contained 302 305, 31 640, and 66 055 slices from the corresponding subsets, yielding exactly 400 000 slices. CT-1 M contained 755 762, 79 099, and 165 139 slices, respectively, yielding exactly 1000 000 slices. The datasets were constructed hierarchically such that CT-0.01 M ⊂ CT-0.4 M ⊂ CT-1 M ⊂ CT-3 M. Thus, all scales retained approximately the same source proportions as CT-3 M, allowing pretraining scale to be varied while controlling the source composition of the CT data. CT-0.01 M provided a size-matched comparison with PET-0.01 M, whereas CT-1 M provided an intermediate point between CT-0.4 M and CT-3 M for characterizing the CT scaling trend.

For the in-domain proximity level, PET pretraining was performed using a cross-dataset strategy between the two finetuning datasets (autoPET and HECKTOR; details in section 2.1.2). Specifically, when one dataset was used for downstream finetuning and evaluation, the other dataset was used exclusively for self-supervised pretraining, and vice versa. This design enables in-domain (PET-to-PET) representation learning while maintaining strict dataset-level separation to prevent information leakage.

For preprocessing, all volumetric CT volumes underwent resampling to 1 mm isotropic resolution with subsequent axial slice extraction. Mammography images, acquired as native 2D projections, were standardized to 1 mm in-plane resolution through padding or center-cropping to preserve anatomical geometry.

#### 2.1.2. Finetuning dataset

To ensure a comprehensive set of downstream tasks for evaluating the impact of pretraining domains, two publicly available whole-body PET/CT datasets featuring diverse lesion types and PET radiotracers were selected for finetuning domain-specialized segmentation models. Both autoPET and HECKTOR datasets underwent standardized preprocessing through the nnU-Net v2 2D configuration pipeline. Volumetric PET/CT data were resampled in-plane to the cohort-specific median resolution using cubic (image) and nearest-neighbor (label) interpolations, followed by axial slice extraction and spatial standardization to 400×400 (autoPET) or 512×512 (HECKTOR) voxels. Intensities were standardized using linear min-max normalization to [0,1] for PET and *z*-score normalization following rescaling to [0,1] for CT.

**autoPET** Gatidis *et al* (2022): The autoPET dataset provides a whole-body PET/CT collection with dual-radiotracer characterization, comprising 500 FDG-PET/CT scans (melanoma, lymphoma, lung cancer) and 538 PSMA-PET/CT scans (prostate carcinoma). The dataset exhibits substantial tumor volume heterogeneity (234.19±398.40 cm$ ^{3}$) with manually annotated lesion masks. The FDG-PET acquisition occurred 60 min post-injection of 350 MBq ^18^F-FDG, while PSMA-PET imaging followed 74 min after administration of 246 MBq ^18^F-PSMA or 214 MBq ^68^Ga-PSMA.

**HECKTOR** Oreiller *et al* (2022): The HECKTOR dataset is a public multi-institutional head-and-neck (H&N) FDG PET/CT cohort with manual delineations of the primary tumor and involved lymph nodes. The primary tumors are located in the oropharynx, larynx, or nasopharynx, with mean volume 12.71±17.28 cm^3^, while lymph nodes have mean volume 13.02±18.75 cm^3^.

In our study, we included 245 patients from five institutions (MDA: 166, HGJ: 49, CHUM: 15, HMR: 12, USZ: 3), all treated with radiotherapy. Imaging was acquired using integrated PET/CT systems across centers, including GE Healthcare Discovery series scanners (HGJ, HMR, CHUM, MDA, USZ). Acquisition protocols varied across sites: administered FDG dose ranged approximately from 315–584 MBq at HGJ, 315–859 MBq at HMR, 315–3182 MBq at CHUM, 327–266 MBq at MDA, and 178–513 MBq at USZ. Uptake times were typically around 90 min (HGJ, HMR, CHUM, MDA) and approximately 60 min at USZ.

### 2.2. Domain-specific pretraining with DINOv3

To adapt DINOv3 to medical PET/CT data, we employed a three-stage self-supervised pretraining pipeline that gradually strengthens semantic consistency, dense structural representation, and multi-scale robustness before downstream finetuning. Rather than relying on a single SSL objective, our approach combines several complementary contemporary losses in a sequential manner. Specifically, the pretraining objective combines four complementary terms: $L_{\mathrm{DINO}}$, which enforces consistency between student and teacher predictions across global and local views; $L_{\mathrm{iBOT}}$, which supervises masked patch prediction to preserve patch-level information (Zhou *et al* 2022); $L_{\mathrm{KoLeo}}$, which promotes dispersion in the representation space (Sablayrolles *et al* 2018); and $L_{\mathrm{Gram}}$, which aligns patch-token similarity structure between teacher and student features (Gatys *et al* 2016). The three-stage pretraining objectives are as follow:

**Stage 1** performs standard domain adaptation from the original natural-image DINOv3 initialization using three losses: the DINO global-local self-distillation loss, $L_{\mathrm{DINO}}$, the iBOT masked patch prediction loss, $L_{\mathrm{iBOT}}$, and the KoLeo feature-uniformity regularizer, $L_{\mathrm{KoLeo}}$. The objective is

\begin{align*} L_{\mathrm{Stage\,1}} = L_{\mathrm{DINO}} + L_{\mathrm{iBOT}} + 0.1\,L_{\mathrm{KoLeo}}.\end{align*}

**Stage 2** augments this objective with a Gram-matrix structural consistency loss, $L_{\mathrm{Gram}}$, applied to patch-level features. This term encourages the student network to preserve the teacher’s second-order patch-token similarity structure, thereby improving dense representation quality while retaining semantic discrimination. The resulting objective is

\begin{align*} L_{\mathrm{Stage\,2}} = L_{\mathrm{DINO}} + L_{\mathrm{iBOT}} + 0.1\,L_{\mathrm{KoLeo}} + 2.0\,L_{\mathrm{Gram}}.\end{align*}

**Stage 3** keeps the same family of losses but adapts training to a mixture of low- and high-resolution crops in order to learn more stable multi-scale representations for high-resolution downstream PET/CT segmentation. In this stage, the Gram loss weight is reduced and the local DINO term is down-weighted relative to Stage 2, yielding

\begin{align*} L_{\mathrm{Stage\,3}} = L_{\mathrm{DINO}} + L_{\mathrm{iBOT}} + 0.1\,L_{\mathrm{KoLeo}} + 1.5\,L_{\mathrm{Gram}}.\end{align*}

**Mathematical formulation** Let $f_\mathrm{s}$ and $f_\mathrm{t}$ denote the student and teacher networks, respectively, where the teacher parameters are updated by exponential moving average (EMA) of the student parameters. For each input image x, we sample two global crops $\{x^{(g_1)}, x^{(g_2)}\}$ and K local crops $\{x^{(l_1)}, \ldots, x^{(l_K)}\}$.

For the DINO self-distillation objective, the student and teacher produce CLS-token logits

\begin{align*} z_\mathrm{s}^{\left(g_i\right)} &amp; = f_\mathrm{s}^{\,\, \mathrm{cls}}\left(x^{\left(g_i\right)}\right), \qquad z_\mathrm{s}^{\left(l_j\right)} = f_\mathrm{s}^{\,\, \mathrm{cls}}\left(x^{\left(l_j\right)}\right),\end{align*}

\begin{align*} z_\mathrm{t}^{\left(g_i\right)} &amp; = f_\mathrm{t}^{\,\, \mathrm{cls}}\left(x^{\left(g_i\right)}\right),\end{align*}

which are used to enforce consistency between student predictions on global and local views and teacher predictions on global views. Let $\sigma(\cdot)$ denote the softmax function and $\mathrm{CE}(\cdot,\cdot)$ the cross-entropy loss. The global and local components are

\begin{align*} L_{\mathrm{DINO}}^{\mathrm{global}} &amp; = \frac{1}{N_g\left(N_g-1\right)} \sum_{i = 1}^{N_g} \sum_{\mathop{j = 1}{\mathop{j \neq i}}}^{N_g} \mathrm{CE}\!\left(\sigma\left(z_\mathrm{t}^{\left(g_j\right)}\right), \sigma\left(z_\mathrm{s}^{\left(g_i\right)}\right)\right),\end{align*}

\begin{align*} L_{\mathrm{DINO}}^{\mathrm{local}} &amp; = \frac{1}{N_l N_g} \sum_{i = 1}^{N_l} \sum_{j = 1}^{N_g} \mathrm{CE}\!\left(\sigma\left(z_\mathrm{t}^{\left(g_j\right)}\right), \sigma\left(z_\mathrm{s}^{\left(l_i\right)}\right)\right),\end{align*}

and the total DINO loss is

\begin{align*} L_{\mathrm{DINO}} = \alpha_{\mathrm{g}} L_{\mathrm{DINO}}^{\mathrm{global}} + \alpha_{\mathrm{l}} L_{\mathrm{DINO}}^{\mathrm{local}},\end{align*}

where $\alpha_{\mathrm{g}}$ and $\alpha_{\mathrm{l}}$ denote the effective weights applied to the global and local terms.

For the iBOT masked image modeling objective, let $\mathcal{M}$ denote the set of masked patch indices, and let

\begin{align*} h_\mathrm{s}\left(u\right) = f_\mathrm{s}^{\,\, \mathrm{mask}}\left(x\right)_u, \qquad h_\mathrm{t}\left(u\right) = f_\mathrm{t}^{\,\, \mathrm{mask}}\left(x\right)_u, \qquad u \in \mathcal{M},\end{align*}

be the student and teacher masked patch predictions. The iBOT loss is

\begin{align*} L_{\mathrm{iBOT}} = \frac{1}{|\mathcal{M}|} \sum_{u \in \mathcal{M}} \mathrm{CE}\!\left(\sigma\left(h_\mathrm{t}\left(u\right)\right), \sigma\left(h_\mathrm{s}\left(u\right)\right)\right).\end{align*}

For KoLeo regularization, let $v_\mathrm{s}^{(g_i)}$ denote the pre-head CLS representation of the ith global crop. The KoLeo term promotes dispersion of these global representations in feature space:

\begin{align*} L_{\mathrm{KoLeo}} = -\frac{1}{N_g} \sum_{i = 1}^{N_g} \log \left( \min_{j \neq i} \left\| v_\mathrm{s}^{\left(g_i\right)} - v_\mathrm{s}^{\left(g_j\right)} \right\|_2 \right).\end{align*}

For structural consistency, let

\begin{align*} F_\mathrm{s} = f_\mathrm{s}^{\,\, \mathrm{patch}}\left(x\right), \qquad F_\mathrm{t} = f_\mathrm{t}^{\,\, \mathrm{patch}}\left(x\right),\end{align*}

denote the student and teacher patch-level feature matrices, where $F_\mathrm{s}, F_\mathrm{t} \in \mathbb{R}^{P \times D}$, P is the number of patches, and D is the feature dimension. These patch-level features are used only for the structural regularization term. We form similarity matrices

\begin{align*} S_\mathrm{s} = F_\mathrm{s} F_\mathrm{s}^\top, \qquad S_\mathrm{t} = F_\mathrm{t} F_\mathrm{t}^\top,\end{align*}

with optional $\ell_2$-normalization of patch features before similarity computation, and define the Gram-based structural consistency loss as

\begin{align*} L_{\mathrm{Gram}} = \mathrm{MSE}\left(S_\mathrm{s}, S_\mathrm{t}\right).\end{align*}

#### 2.2.1. Implementation details

The DINOv3 self-distillation framework maintained the teacher network parameters were updated via an EMA of the student parameters with an EMA decay starting at 0.996 and linearly increasing to 1.0 by the end of each stage. Post-training for further alignment in x-ray, CT, and PET domain was initialized from the DINOv3 vision transformer base (ViT-B) checkpoint pretrained on LVD-1689 M. In **Stage 1**, the student network was trained for 1000 epochs using AdamW with a learning rate linearly warmed up over the first 100 epochs to a peak value of $2\times10^{-4}$, after which it was kept constant. Weight decay was fixed at 0.04 and AdamW momentum parameters were $(\beta_1,\beta_2) = (0.9,0.98)$. The teacher network was updated as an EMA of the student with momentum 0.994. The total batch size was 512 distributed on two NVIDIA A100 GPUs.

In **Stage 2**, a secondary Gram teacher was initialized from the EMA teacher checkpoint at epoch 200 of Stage 1. The student encoder was trained for an additional 100 epochs using AdamW with a constant learning rate of $5\times10^{-5}$ and weight decay 0.04, while the Gram teacher was updated as an EMA of the student with momentum 0.999.

In **Stage 3**, the student and teacher networks were initialized from the final Stage 2 checkpoints. The student was trained for an additional 100 epochs using AdamW with a learning rate linearly warmed up from $1\times10^{-6}$ to $2.5\times10^{-5}$ over the first 3 epochs, followed by cosine decay to zero, while the teacher was updated as an EMA of the student with momentum 0.999.

### 2.3. Specialized finetuning on downstream tasks

We selected nnU-Net framework as our supervised baseline due to its established state-of-the-art for supervised medical image segmentation. To assess the effect of domain-specific pretraining, we replaced the standard nnU-Net encoder with a ViT-based encoder initialized from the frozen DINOv3 weights. The resulting patch embeddings generated by frozen DINOv3 was then decoded into dense segmentation maps using the Primus decoder (Wald *et al* 2025). The Primus decoder consisted of several ConvTranspose2d upsampling modules, each followed by LayerNorm and GeLU activations. This architecture systematically halves the channel dimension at each step, and terminates with a 1×1 convolution to produce the C-class segmentation logits. This design provides a computationally efficient alternative to CNN decoders by directly inverting the ViT patch embedding process, thus is significantly more lightweight than U-Net–style CNN decoders. Each model was trained for 1000 epochs using the AdamW optimizer with $(\beta_1,\beta_2) = (0.9, 0.98)$, a base learning rate of $3\times10^{-4}$, weight decay of $5\times10^{-2}$, cosine learning rate decay, and linear warmup during the first 50 epochs. Model training uses Dice-based loss computed at the slice level, assigning equal weight to each slice. Batch size was set to 16 for the AutoPET dataset and 12 for the HECKTOR dataset, as automatically determined by the nnU-Net planning pipeline. Each fold was trained on a single NVIDIA A100 GPU. The training time was approximately 92 s/epoch for AutoPET and 23 s/epoch for HECKTOR, with GPU memory usage of about 15 GB and 17 GB, respectively. Under these settings, each fold required approximately 25 h for AutoPET and 6 h for HECKTOR to complete.

### 2.4. Evaluation

#### 2.4.1. Model configurations

Here, the progression of model configurations is designed to evaluate two interacting factors: (i) domain proximity to the downstream PET/CT segmentation task and (ii) the scale of the pretraining dataset. We evaluated six encoder initialization variants under the same nnU-Net segmentation framework using the same overall segmentation architecture, including the same ViT-based encoder and Primus decoder, differing only in encoder initialization. Thus, decoder design, model capacity, optimization settings, and training protocol were controlled across all experiments. The from-scratch model was initialized with random weights and served as the supervised baseline. The natural-image model used the original DINOv3 encoder, while the x-ray, CT, and PET models used DINOv3 encoders initialized from the natural-image pretrained model and further post-trained on mammography, CT, or PET images. The primary CT models were CT-0.4 M and CT-3 M; CT-0.4 M was selected to match the x-ray pretraining condition, enabling a size-controlled comparison between x-ray-0.4 M and CT-0.4 M. The CT-3 M model used the larger CT pretraining collection and therefore evaluates the additional effect of CT data scale and anatomical diversity. CT-0.01 M was included for autoPET to provide a size-matched comparison with PET-0.01 M. CT-1 M was included for HECKTOR as an intermediate CT scale point. The CT-1 M, CT-0.4 M, and CT-0.01 M model were pretrained by applying the same three-stage self-supervised DINOv3 post-training pipeline used for CT-3 M, but restricting to a subset of the CT-3 M pretraining pool. All downstream finetuning experiments used the same data augmentations, loss functions, optimizer settings, and training schedule to ensure that differences in performance were attributable solely to the pretraining domain and available dataset size. Architectural and initialization details for these model variants are summarized in table 1.

**Table 1.** Summary of comparative models. All models were trained with the nnU-Net v2 framework using identical downstream configurations; model variants differed only in encoder initialization and the dataset used for self-supervised post-training. Slash-separated entries in the pretraining data size column indicate the distinct pretraining scales evaluated for that model family.

Model name	Encoder initialization	Pretraining source and configuration	Pretraining data size	Domain proximity	Downstream configuration
From-scratch	Random initialization	No self-supervised pretraining	none	–	Standard supervised nnU-Net trained from scratch

Natural-image	Frozen DINOv3	Original DINOv3 encoder pretrained on natural images	1689 M	Low	Frozen encoder with identical Primus decoder and nnU-Net training protocol

x-ray	Frozen x-ray DINOv3	Natural-image DINOv3 further post-trained on mammography images using the DINOv3 self-supervised pipeline	0.4 M	Medium	Frozen encoder with identical Primus decoder and nnU-Net training protocol

CT	Frozen CT DINOv3	Natural-image DINOv3 further post-trained on nested CT subsets using the same three-stage self-supervised pipeline as CT-3 M	0.01 / 0.4 / 1 / 3 M	High	Frozen encoder with identical Primus decoder and nnU-Net training protocol

PET	Frozen PET DINOv3	Natural-image DINOv3 further post-trained on PET images; the PET pretraining set depended on the downstream dataset to maintain dataset-level separation	0.01 / 0.3 M	In-domain	Frozen encoder with identical Primus decoder and nnU-Net training protocol

#### 2.4.2. Cross-validation protocol and statistical testing

Experimental results were obtained on two downstream PET/CT tumor segmentation tasks using a five-fold stratified cross-validation protocol. In each fold, one subset was used for testing and the remaining subsets for training and validation, with results averaged across all five folds. All reported metrics are calculated using the mean performance across the five held-out folds.

Paired two-sided Wilcoxon signed-rank tests were performed on subject-level Dice scores. Holm correction was applied separately within each dataset-target endpoint family across all 15 unique pairwise comparisons among the six primary comparison models (from-scratch, natural-image, x-ray, CT-0.4 M, PET, and CT-3 M). The additional CT-scale ablation points (CT-1 M for HECKTOR and CT-0.01 M for autoPET) were summarized descriptively to characterize the scale trend and were not included in the Holm-corrected primary comparison family. For each family, raw *p*-values were ordered from smallest to largest and compared against the rank-dependent Holm threshold α/(m−r+1), where α=0.05, m=15, and r is the ordered rank. Following the Holm step-down procedure, once a comparison failed to meet its rank-dependent threshold, that comparison and all subsequent lower-ranked comparisons in the same family were treated as non-rejected. The main manuscript reports exact Holm-adjusted *p*-values for central comparisons, and the complete ordered Holm sequences, including raw *p*-values, rank, critical threshold, adjusted *p*-value, and rejection decision, are reported in supplementary table 1.

#### 2.4.3. Evaluation metrics

Dice score was calculated as the unweighted mean of subject-level Dice scores and is therefore reported as mean (standard deviation) across subjects. For lesion-level assessment, we evaluated connected-component detection using the IoU-based one-to-one matching rule corresponding to Criterion 2 of Ahamed *et al* (2023). Ground-truth and predicted masks were decomposed into three-dimensional connected components using 26-connectivity, meaning that foreground voxels were assigned to the same component when they touched through a face, edge, or corner within the $3 \times 3 \times 3$ voxel neighborhood. For each subject, we computed the pairwise component IoU matrix between all predicted and ground-truth components and solved a bipartite assignment that maximized total IoU. A matched predicted-ground-truth pair was counted as a true-positive lesion only when its IoU was ${\unicode{x2A7E}} 0.50$. Matched pairs below this threshold were discarded; unmatched ground-truth components were counted as false negatives, and unmatched predicted components were counted as false positives. This one-to-one assignment explicitly resolves many-to-one fragmentation and one-to-many fusion cases by allowing each predicted component and each ground-truth component to participate in at most one match. Lesion-level sensitivity, precision, and *F*1 score were computed as $\mathrm{TP}_\mathrm{lesion}/(\mathrm{TP}_\mathrm{lesion}+\mathrm{FN}_\mathrm{lesion})$, $\mathrm{TP}_\mathrm{lesion}/(\mathrm{TP}_\mathrm{lesion}+\mathrm{FP}_\mathrm{lesion})$, and $2 \times \mathrm{precision} \times \mathrm{sensitivity}/(\mathrm{precision}+\mathrm{sensitivity})$, respectively. PSMA and FDG scans in autoPET were evaluated as separate binary lesion-detection tasks. To further examine performance as a function of lesion size, tumor volumes were stratified into three categories using dataset-specific thresholds corresponding to an approximately even split into tertiles. For the autoPET dataset, PSMA lesions were categorized as small ($\unicode{x2A7D}$10.4561 cm$ ^3$), medium (>10.4561 and $\unicode{x2A7D}$142.0260 cm$ ^3$), and large (>142.0260 cm$ ^3$), while FDG lesions were categorized as small ($\unicode{x2A7D}$45.7125 cm$ ^3$), medium (>45.7125 and $\unicode{x2A7D}$191.6581 cm$ ^3$), and large (>191.6581 cm$ ^3$). For the HECKTOR dataset, primary tumors (GTVp) were categorized as small ($\unicode{x2A7D}$5.5572 cm$ ^3$), medium (>5.5572 and $\unicode{x2A7D}$12.6622 cm$ ^3$), and large (>12.6622 cm$ ^3$), while lymph nodes (GTVn) were categorized as small ($\unicode{x2A7D}$7.3337 cm$ ^3$), medium (>7.3337 and $\unicode{x2A7D}$17.9813 cm$ ^3$), and large (>17.9813 cm$ ^3$).

#### 2.4.4. Qualitative feature visualization

To qualitatively examine the learned representation space, we performed PCA on the patch-level feature matrix $F_\mathrm{s} \in \mathbb{R}^{P \times D}$ produced by the pretrained DINOv3 encoder, where P is the number of spatial patches and D is the feature dimension. For each input slice, the first three principal components of the patch-level features were retained, reshaped to the spatial patch grid, and interpolated to image resolution to generate RGB visualizations. These PCA maps were used only for qualitative analysis of the learned feature and were not involved in training or quantitative evaluation.

## 3. Results

### 3.1. Impact of pretraining domain on PET/CT tumor segmentation performance

#### 3.1.1. Effect of pretraining domain and dataset scale on segmentation performance

Systematic evaluation across all target regions and PET modalities revealed clear performance differences across pretraining strategies (table 2). CT-based pretraining showed the most consistent overall performance: CT-3 M achieved the best performance for HECKTOR GTVn and autoPET FDG, while CT-0.4 M was best for autoPET PSMA. Compared with the from-scratch baseline, CT-3 M yielded the largest gain in autoPET FDG, improving Dice from 0.624 to 0.714 (+9 points, Holm-adjusted p<0.001), and increased Dice for HECKTOR GTVp from 0.501 to 0.581 (+8 points, Holm-adjusted p=0.001 50). These gains suggest that CT-domain pretraining was most useful when anatomical context and lesion-boundary localization both contributed strongly to the segmentation task.

**Table 2.** Performance comparison across pretraining domains. Results are reported as mean (standard deviation). Dice is computed as the unweighted average of subject-level Dice scores. Sens., Prec., and *F*1 denote lesion-level sensitivity, precision, and *F*1 score under IoU-maximizing one-to-one connected-component matching with IoU ${\unicode{x2A7E} }0.50$, respectively. Best results for each target and metric are highlighted in **bold**.

	*Pretraining domain and data size*		From-scratch	Natural-1.7B	x-ray-0.4 M	CT-0.4 M	CT-1 M	CT-3 M	PET-0.3 M
HECKTOR	GTVp	Dice	0.501 (0.24)	0.513 (0.22)	0.485 (0.23)	0.550 (0.22)	0.567 (0.23)	0.581 (0.25)	**0.598 (0.21)**
		Sens.	0.744 (0.27)	0.752 (0.26)	0.731 (0.27)	0.781 (0.25)	0.792 (0.24)	0.806 (0.25)	**0.821 (0.24)**
		Prec.	0.632 (0.29)	0.641 (0.28)	0.608 (0.29)	0.681 (0.27)	0.695 (0.27)	0.714 (0.27)	**0.738 (0.26)**
		*F*1	0.683 (0.28)	0.692 (0.27)	0.664 (0.28)	0.728 (0.25)	0.741 (0.25)	0.757 (0.25)	**0.777 (0.24)**

	GTVn	Dice	0.616 (0.26)	0.515 (0.26)	0.578 (0.26)	0.546 (0.22)	0.584 (0.23)	**0.627 (0.25)**	0.592 (0.25)
		Sens.	0.566 (0.30)	0.491 (0.32)	0.544 (0.31)	0.517 (0.30)	0.559 (0.29)	**0.584 (0.28)**	0.563 (0.29)
		Prec.	0.431 (0.32)	0.362 (0.33)	0.408 (0.31)	0.387 (0.30)	0.423 (0.29)	**0.446 (0.29)**	0.426 (0.30)
		*F*1	0.489 (0.31)	0.417 (0.32)	0.466 (0.30)	0.443 (0.29)	0.482 (0.28)	**0.506 (0.28)**	0.485 (0.29)

	*Pretraining domain and data size*		From-scratch	Natural-1.7B	x-ray-0.4 M	CT-0.01 M	CT-0.4 M	CT-3 M	PET-0.01 M

autoPET	PSMA	Dice	0.524 (0.29)	0.483 (0.30)	0.513 (0.29)	0.327 (0.25)	**0.585 (0.26)**	0.565 (0.29)	0.394 (0.29)
		Sens.	0.739 (0.21)	0.704 (0.22)	0.728 (0.21)	0.571 (0.23)	**0.801 (0.17)**	0.777 (0.19)	0.622 (0.23)
		Prec.	0.603 (0.22)	0.558 (0.23)	0.590 (0.22)	0.421 (0.22)	**0.681 (0.19)**	0.654 (0.20)	0.472 (0.22)
		*F*1	0.664 (0.21)	0.623 (0.22)	0.652 (0.21)	0.485 (0.22)	**0.736 (0.18)**	0.710 (0.19)	0.536 (0.22)

	FDG	Dice	0.624 (0.26)	0.533 (0.29)	0.655 (0.25)	0.410 (0.28)	0.683 (0.23)	**0.714 (0.23)**	0.339 (0.25)
		Sens.	0.764 (0.18)	0.692 (0.21)	0.791 (0.16)	0.612 (0.23)	0.817 (0.15)	**0.846 (0.14)**	0.548 (0.23)
		Prec.	0.641 (0.19)	0.548 (0.22)	0.682 (0.17)	0.456 (0.21)	0.716 (0.16)	**0.751 (0.15)**	0.392 (0.20)
*F*1		0.697 (0.18)	0.612 (0.21)	0.733 (0.16)	0.523 (0.21)	0.763 (0.15)	**0.796 (0.14)**	0.457 (0.20)

Because at least three ordered scale points are needed to support a directional claim about CT pretraining scale, the CT comparison included CT-0.4 M, CT-1 M, and CT-3 M for HECKTOR, and CT-0.01 M, CT-0.4 M, and CT-3 M for autoPET. HECKTOR showed a monotonic CT scaling pattern for both primary tumors (0.550, 0.567, and 0.581) and nodal lesions (0.546, 0.584, and 0.627), supporting a scale-dependent performance effect in this dataset. In autoPET, however, the CT scaling pattern was target-dependent: FDG increased across all three CT scales (0.410, 0.683, and 0.714), whereas PSMA increased sharply from CT-0.01 M to CT-0.4 M but then decreased slightly at CT-3 M (0.327, 0.585, and 0.565). Thus, larger CT pretraining scale improved performance for some endpoints but did not produce a uniform monotonic response across all targets.

The matched x-ray-0.4 M versus CT-0.4 M comparison further separated domain proximity from pretraining dataset size. At the same nominal pretraining size, CT-0.4 M outperformed x-ray-0.4 M for three of the four endpoints, with the clearest separation observed for autoPET PSMA (0.585 vs 0.513). This pattern supports a CT-domain effect independent of the original CT-3 M scale advantage. The remaining HECKTOR GTVn endpoint favored x-ray-0.4 M (0.546 vs 0.578), indicating that domain proximity alone did not determine performance and that target-specific anatomy also influenced transfer.

PET pretraining showed the strongest evidence of target- and scale-dependent behavior. In HECKTOR, where the PET-pretrained model used approximately 0.3 million samples, PET produced the highest observed GTVp Dice (0.598), but its small margin over CT-3 M (0.581) was not significant after Holm correction; therefore, no statistical ordering between PET and CT-3 M is inferred for this endpoint. For HECKTOR GTVn, PET-0.3 M (0.592) fell between CT-1 M (0.584) and CT-3 M (0.627), suggesting that the CT-equivalent pretraining scale for this target lies between 1 M and 3 M CT slices under the present setup. This difference between GTVp and GTVn suggests that primary-tumor segmentation may rely more on PET uptake patterns, whereas nodal segmentation may rely more on broader CT anatomical context and pretraining diversity.

HECKTOR GTVn was also the only endpoint where the from-scratch baseline was competitive with the top pretrained model, with Dice close to CT-3 M (0.616 vs 0.627). This pattern suggests that supervised nnU-Net training alone captured much of the information needed for this nodal target, although CT-3 M still provided the highest observed performance.

In autoPET, where PET pretraining used a smaller dataset of approximately 0.01 million samples, PET-0.01 M remained below the larger CT models for both PSMA and FDG. At the size-matched 0.01 M scale, however, PET and CT showed different ordering by target: PET-0.01 M exceeded CT-0.01 M for PSMA (0.394 vs 0.327), whereas CT-0.01 M exceeded PET-0.01 M for FDG (0.410 vs 0.339). This divergence indicates that in-domain PET pretraining at very small scale had target-dependent rather than uniform effects across autoPET radiotracers.

Lower-proximity pretraining provided less consistent improvements. Natural-image pretraining did not consistently improve over the from-scratch baseline, and x-ray-0.4 M improved over natural-image pretraining only for selected endpoints after Holm correction. The IoU-matched lesion-level metrics in table 2 provide a complementary instance-level assessment of missed lesions and false-positive predicted components using the stricter Criterion 2 detection threshold of IoU ${\unicode{x2A7E}} 0.50$. These instance-level trends are directionally consistent with the representative qualitative examples in figure 2, where the CT-pretrained model more closely follows the ground-truth contours and shows fewer visible spurious regions than the comparator models. Because figure 2 shows selected representative cases rather than cohort-level statistics, it was used only as qualitative support for the metric trends. Overall, CT-based pretraining provided robust performance across datasets by combining domain relevance with sufficient pretraining data scale, whereas PET pretraining depended more strongly on the available PET pretraining scale and target-specific imaging characteristics.

**Figure 2.**
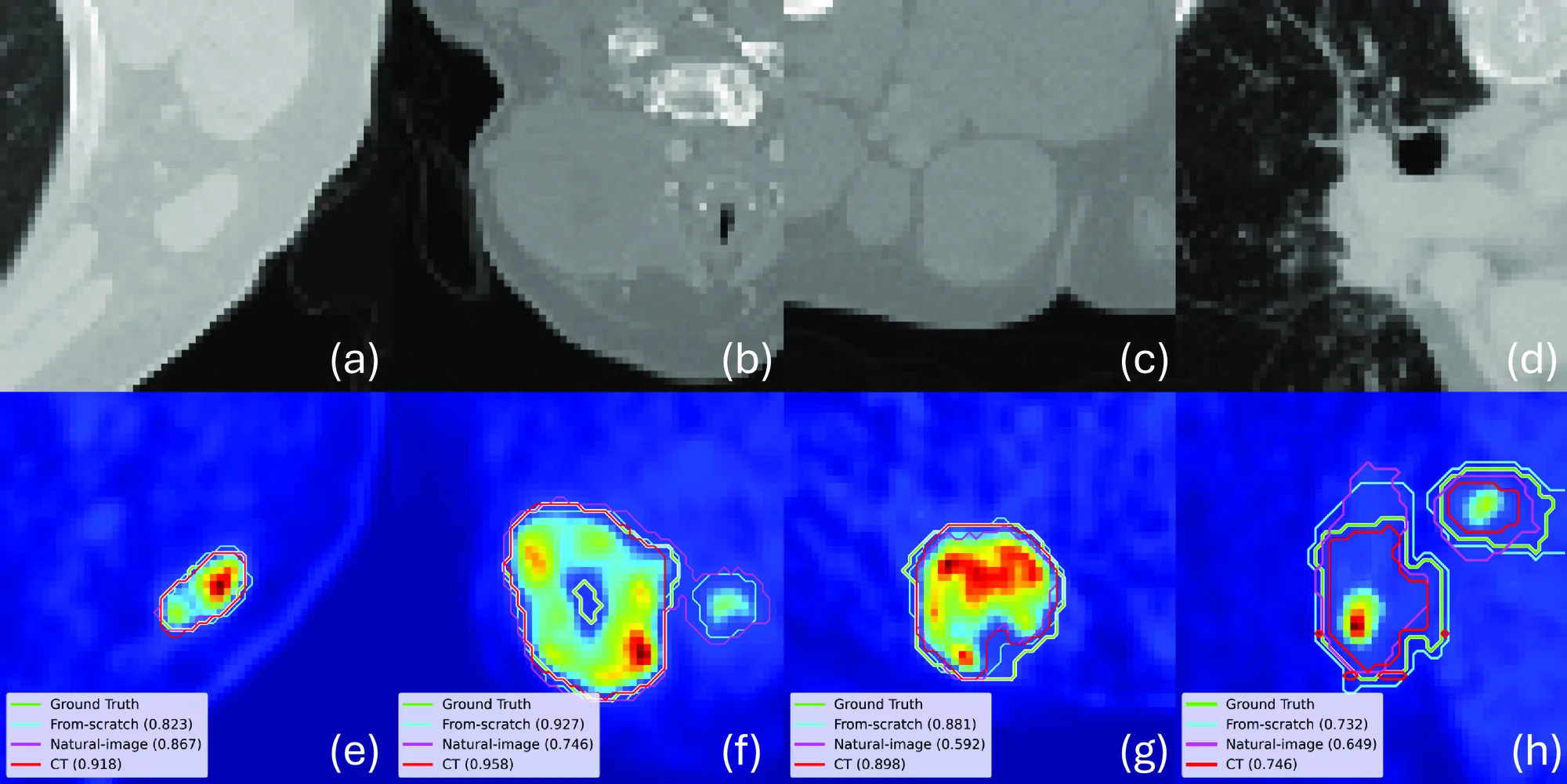
Qualitative comparison of segmentation performance across tumor volume subgroups. Representative cases from the autoPET dataset demonstrate segmentation outcomes for (a), (e) small-volume melanoma lesion (5.64 cm^3^), (b), (f) medium-volume lymphoma lesion (53.65 cm^3^), (c), (g) large-volume melanoma lesion (133.6 cm^3^), and (d), (h) a medium-volume lung lesion (116.9 cm^3^) selected to be closer to cohort-average performance and to broaden the qualitative comparison across initialization strategies. Each panel displays co-registered CT/PET images with ground-truth contours and model predictions. Among them, the CT DINOv3 model demonstrates improved boundary delineation and reduced false-positive regions across all volume categories.

### 3.2. Segmentation performance stratified by tumor volume

When stratified by tumor volume across both datasets (tables 4 and 5), distinct performance patterns were observed across models. The CT model outperformed the from-scratch model across most volume groups and targets, with statistically significant improvements (*p* <0.001), except for H&N lymph nodes where a lower significance level was observed (*p* <0.05). In addition, the CT model generally exhibited lower standard deviations compared to the from-scratch model, indicating reduced variability across subjects, except again for H&N lymph nodes. Incremental improvements from natural-image to x-ray to CT were observed in most volume groups.

The PET-pretrained model exhibits different behavior depending on target and lesion size. In HECKTOR, PET pretraining improves over the x-ray model for GTVp targets across all volume groups (small: 0.424 vs 0.340; medium: 0.614 vs 0.486; large: 0.741 vs 0.632), but remains below the CT model (e.g. large: 0.741 vs 0.799). For GTVn targets, PET pretraining does not consistently improve over x-ray and remains below CT across all volume groups.

In autoPET, PET pretraining results in lower performance across all volume groups. For PSMA, Dice is 0.179, 0.413, and 0.595 for small, medium, and large lesions, respectively, compared to 0.386, 0.655, and 0.741 for CT. For FDG, PET pretraining yields 0.239, 0.361, and 0.422 across increasing volume groups, compared to 0.636, 0.780, and 0.825 for CT. These differences are statistically significant across comparisons (*p* <0.001).

Across all models, segmentation performance decreases with decreasing tumor volume, a trend consistently observed across datasets and targets. Qualitative examples in figure 2 further illustrate these patterns. Smaller lesions are associated with increased variability across models, whereas larger lesions tend to exhibit mild over-segmentation beyond the ground-truth contours. In addition, figure 2 highlights differences in model behavior with respect to false-positive predictions. In tile (f), the additional right-lateral focus of high PET uptake corresponds to physiologic bowel activity in an adjacent bowel loop rather than a ground-truth lymphoma lesion. This focus was incorrectly segmented by the from-scratch and natural-image pretrained models, likely because focal bowel uptake can have intensity and local appearance similar to malignant FDG-avid disease on PET alone. In contrast, the CT-pretrained model excluded this region and better matched the ground-truth annotation, suggesting that CT-domain pretraining helped the model use anatomic context to distinguish physiologic bowel uptake from tumor.

### 3.3. PCA visualization of learned feature maps

The PCA visualizations revealed clear modality-dependent differences in the learned feature organization (figure 3). For CT inputs, the feature maps aligned closely with anatomical structures, showing pronounced responses along tissue boundaries and intensity transitions. This suggests that the pretrained encoder captured structural priors that are relevant for downstream segmentation.

**Figure 3.**
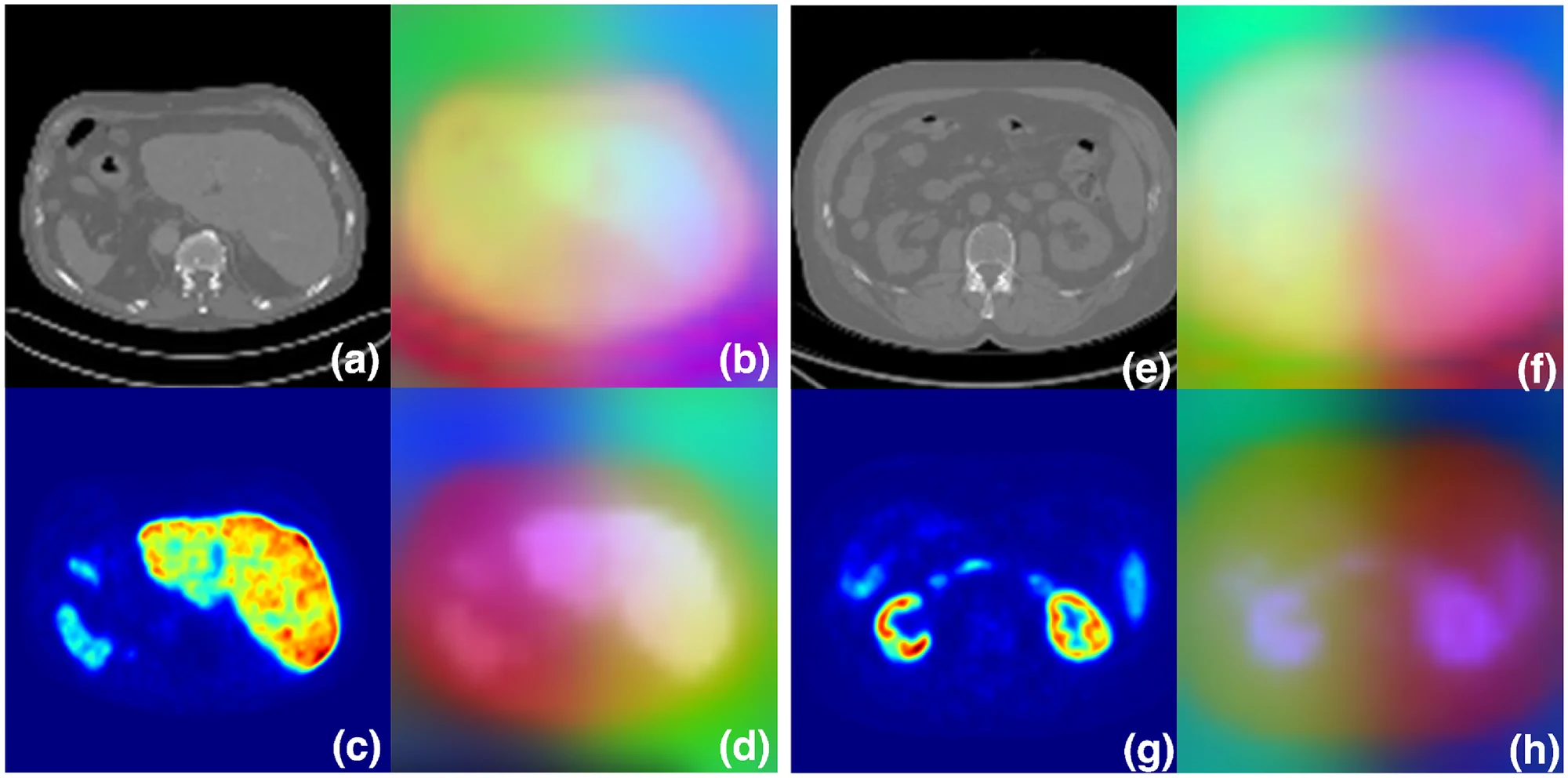
Principal component analysis of feature representations from CT DINOv3 encoder. (a), (e) Input CT images; (b), (f) Corresponding PCA projections of CT features; (c), (g) Co-registered PET images; (d), (h) PCA projections of PET features. Feature activations align with anatomical boundaries in CT and physiological uptake regions in PET, demonstrating the encoder’s sensitivity to structural patterns while revealing limitations in capturing focal metabolic activity.

For PET inputs, the feature maps exhibited distinct activation patterns in regions of physiological tracer uptake, particularly around high-uptake organs such as the liver and kidneys. In contrast, some small focal lesions with intense uptake produced weaker or less distinct responses. These qualitative findings suggest that the learned representations are more strongly tuned to structural information than to subtle functional PET-specific signals.

Overall, the PCA maps support the quantitative results by indicating that CT-based DINOv3 pretraining yields robust structural representations, while also highlighting a modality gap between CT-derived structural features and PET functional characteristics.

## 4. Discussion

Medical vision foundation models have emerged as powerful tools for learning transferable representations across diverse imaging tasks. Among them, the self-supervised DINOv3 framework, originally developed for natural images, has demonstrated notable few-shot adaptability across multiple domains, including medical imaging tasks such as CT and MRI segmentation and classification. Despite these successes, its performance in PET imaging remains suboptimal (Liu *et al* 2025). This limitation may be due to the lack of distinct anatomical boundaries and the markedly different spatial resolution in PET, which deviates substantially from the characteristics of natural images. Recently, MedDINOv3 (Li *et al* 2025) was introduced, adopting the same SSL paradigm as DINOv3 and subsequently undergoing post-training on large-scale CT datasets. Motivated by this, our study was designed to address two central questions: (1) whether large-scale self-supervised pretraining provides a more effective dense feature extractor than task-specific supervised learning, and (2) whether domain proximity between the pretraining and downstream datasets influences downstream performance after accounting for pretraining dataset scale.

To examine the first question, we compared the standard nnU-Net trained from random initialization (from-scratch model) with variants in which its encoder was replaced by frozen DINOv3 encoders pretrained on medical imaging data. The Results show that self-supervised pretraining was not uniformly advantageous, but that domain-relevant pretraining could improve downstream segmentation when paired with sufficient pretraining scale. This pattern suggests that pretraining on abundant unlabeled medical images can yield feature representations that are more transferable and robust than those learned only from the annotated downstream training folds. The PCA feature maps in figure 3 provide qualitative support for this interpretation by showing that CT-pretrained features capture anatomical structural patterns relevant to segmentation.

The matched-scale and multiscale pretraining design separates two related but distinct factors: similarity between the pretraining domain and the downstream PET/CT task, and the amount/diversity of unlabeled data used during pretraining. The resulting pattern indicates that domain proximity is important but not sufficient on its own. Downstream segmentation performance is better understood as the joint outcome of imaging-domain proximity, pretraining dataset scale, pretraining diversity, and target-specific imaging characteristics.

The different behavior of HECKTOR primary tumors and nodal targets provides a useful example of this interaction. Primary-tumor segmentation may depend more strongly on focal PET uptake patterns, whereas nodal segmentation may rely more on broader CT anatomical context and pretraining diversity. The competitive from-scratch performance for HECKTOR GTVn suggests that supervised nnU-Net training alone captured much of the information needed for this nodal target. One possible explanation is that nodal targets in the HECKTOR cohort may have more constrained spatial and morphological patterns than whole-body autoPET lesions, allowing the supervised baseline to learn a strong target-specific representation from the training folds. These findings are consistent with prior work showing that domain-specific self-supervised pretraining can improve medical-image transfer and robustness (Azizi *et al* 2021), and with broader robustness studies showing that larger and more diverse training distributions can improve robustness to natural distribution shifts (Taori *et al* 2020).

Compared with prior foundation model studies for PET/CT segmentation, our findings highlight several advantages of the proposed approach. The quantitative results in table 2, the statistical comparisons in table 3, and the qualitative feature visualization in figure 3 show that frozen DINOv3 encoders pretrained on domain-relevant medical images can improve PET/CT lesion segmentation without requiring manual prompts during inference. While prompt-based frameworks such as SAM have achieved competitive results (Ren *et al* 2024, Fang *et al* 2026), they rely on explicit manual inputs (e.g. bounding boxes), introducing a trade-off between automation and supervision. In contrast, the DINOv3 framework operates in a fully self-supervised manner and does not require paired PET–CT inputs or labeled data during pretraining (Oh *et al* 2025); in the present experiments, this design supported fully automatic PET/CT lesion segmentation under the model configurations summarized in table 1.

**Table 3.** Statistical comparisons for Dice performance using Holm-adjusted *p*-values. Statistical significance was assessed using paired two-sided Wilcoxon signed-rank tests on subject-level Dice scores, with Holm correction applied separately within each dataset-target endpoint family across all 15 unique pairwise comparisons among the six primary comparison models. *P*-values below 0.001 are reported as ${\lt} 0.001$, while values at or above 0.001 are reported exactly. The CT-scale ablation points were summarized descriptively in table 2 and were not included in this Holm-corrected primary comparison table. CT-0.4 M comp., PET comp., and CT-3 M comp. indicate comparisons against the corresponding reference model. Boldface indicates that the reference model in that row had the higher observed Dice score.

Dataset	Target	Comparison	From-scratch	Natural-image	x-ray	CT-0.4 M	PET	CT-3 M
HECKTOR	GTVp Dice	CT-0.4 M comp.	**0.00 960**	**0.0140**	${\lt} 0.001$	—	0.0168	0.0900
		PET comp.	**0.00 144**	**0.00 156**	${\lt} 0.001$	**0.0168**	—	**1.000**
		CT-3 M comp.	**0.00 150**	**0.00 156**	${\lt} 0.001$	**0.0900**	1.000	—

	GTVn Dice	CT-0.4 M comp.	0.00 132	**0.270**	0.0480	—	0.00 140	<0.001
		PET comp.	0.0210	${\lt} 0.001$	**0.552**	**0.00 140**	—	0.00 140
CT-3 M comp.		**0.0276**	${\lt} 0.001$	**0.00 140**	${\lt} 0.001$	**0.00 140**	—

autoPET	PSMA Dice	CT-0.4 M comp.	${\lt} 0.001$	${\lt} 0.001$	${\lt} 0.001$	—	${\lt} 0.001$	**0.0240**
		PET comp.	<0.001	<0.001	<0.001	<0.001	—	<0.001
		CT-3 M comp.	${\lt} 0.001$	${\lt} 0.001$	**0.00 163**	0.0240	${\lt} 0.001$	—

	FDG Dice	CT-0.4 M comp.	${\lt} 0.001$	${\lt} 0.001$	**0.00 900**	—	${\lt} 0.001$	0.0240
		PET comp.	<0.001	<0.001	<0.001	<0.001	—	<0.001
CT-3 M comp.		${\lt} 0.001$	${\lt} 0.001$	${\lt} 0.001$	**0.0240**	${\lt} 0.001$	—

Our evaluation also spans a broader range of clinical conditions than previously reported, incorporating multiple public benchmarks, heterogeneous radiotracers, and a wide spectrum of tumor sizes. Notably, earlier studies have reported Dice near 80% on private datasets; however, the absence of tumor volume stratification limits the interpretability of these results. As demonstrated in tables 4 and 5, lesion size exerts a substantial influence on segmentation accuracy, and without controlling for this factor, direct comparison is neither reliable nor meaningful. Our findings underscore the necessity of volume-stratified evaluation when assessing PET/CT foundation models and suggest that reported single-value Dice may obscure important performance variability.

Our study also acknowledges certain limitations. While we incorporated a PET-pretrained DINOv3 variant trained on up to 0.3 million images, this dataset remains substantially smaller than the large-scale datasets used for natural-image (1689 million) and CT pretraining (3.8 million), limiting the ability to fully isolate the effect of PET domain alignment from dataset scale. Furthermore, for the autoPET dataset, the official test set is reserved for the challenge and consists of data from unseen institutions (Dexl *et al* 2025), which is not publicly accessible. As a result, we were unable to benchmark our method against the challenge leaderboard and instead report performance using a rotating five-fold cross-validation on the training set.

**Table 4.** Segmentation performance stratified by tumor volume for the HECKTOR dataset. Primary tumors (GTVp) were categorized as small (*n* = 82; ${\unicode{x2A7D}} 5.5572$ cm$ ^{3}$), medium (*n* = 81; ${\gt} 5.5572$ and ${\unicode{x2A7D}} 12.6622$ cm$ ^{3}$), and large (*n* = 82; ${\gt} 12.6622$ cm$ ^{3}$); lymph nodes (GTVn) were categorized as small (*n* = 82; ${\unicode{x2A7D}} 7.3337$ cm$ ^{3}$), medium (*n* = 81; ${\gt} 7.3337$ and ${\unicode{x2A7D}} 17.9813$ cm$ ^{3}$), and large (*n* = 82; ${\gt} 17.9813$ cm$ ^{3}$). Statistical significance was assessed using paired Wilcoxon signed-rank tests with Holm correction for multiple comparisons. Asterisk annotations (*, **, ***) indicate the level of significance for comparisons between the CT model and all preceding models (* *p* <0.05, ** *p* <0.01, *** *p* <0.001). Letter annotations denote statistically significant differences (*p* <0.05) between successive model configurations: ‘*a*’ indicates the comparison between the from-scratch model and the natural-image model, ‘*b*’ between the natural-image and x-ray model, ‘*c*’ between the x-ray and CT-0.4 M model, and ‘*d*’ between the CT-0.4 M model and PET model. The placement of each letter indicates the higher-performing model within the corresponding comparison.

Model	Pretraining data size	Volume	GTVp Dice	GTVn Dice
From-scratch	none	Small	0.362 (0.215)$ ^{\scriptsize***}$	0.453 (0.281)$ ^{\scriptsize*a}$
		Medium	0.536 (0.198)$ ^{\scriptsize***}$	0.682 (0.231)$ ^{\scriptsize*a}$
		Large	0.646 (0.182)$ ^{\scriptsize***}$	0.719 (0.119)$ ^{\scriptsize*a}$

Natural-image	1689 M	Small	0.311 (0.149)$ ^{\scriptsize***}$	0.262 (0.213)$ ^{\scriptsize***}$
		Medium	0.508 (0.177)$ ^{\scriptsize***}$	0.589 (0.181)$ ^{\scriptsize***}$
		Large	0.644 (0.148)$ ^{\scriptsize***}$	0.613 (0.150)$ ^{\scriptsize***}$

x-ray	0.4 M	Small	0.340 (0.198)$ ^{\scriptsize**}$	0.367 (0.259)$ ^{\scriptsize*}$
		Medium	0.486 (0.202)$ ^{\scriptsize***}$	0.672 (0.177)$ ^{\scriptsize**bc}$
		Large	0.632 (0.162)$ ^{\scriptsize***}$	0.684 (0.168)$ ^{\scriptsize***bc}$

CT	0.4 M	Small	0.363 (0.233)$ ^{\scriptsize***c}$	0.400 (0.206)$ ^{\scriptsize c}$
		Medium	0.625 (0.151)$ ^{\scriptsize***c}$	0.601 (0.199)$ ^{\scriptsize***}$
		Large	0.664 (0.105)$ ^{\scriptsize***c}$	0.638 (0.182)$ ^{\scriptsize***}$

PET	0.3 M	Small	0.424 (0.198)$ ^{\scriptsize***d}$	0.353 (0.276)$ ^{\scriptsize**d}$
		Medium	0.614 (0.174)$ ^{\scriptsize***}$	0.649 (0.195)$ ^{\scriptsize***d}$
		Large	0.741 (0.139)$ ^{\scriptsize d***}$	0.694 (0.146)$ ^{\scriptsize***d}$

CT	3 M	Small	**0.472 (0.224)**	**0.398 (0.283)**
		Medium	**0.684 (0.170)**	**0.705 (0.173)**
		Large	**0.799 (0.135)**	**0.759 (0.104)**

**Table 5.** Segmentation performance stratified by tumor volume for the autoPET dataset. PSMA lesions were stratified as small (*n* = 180; ${\unicode{x2A7D} }10.4561$ cm$ ^{3}$), medium (*n* = 179; ${> }10.4561$ and ${\unicode{x2A7D}} 142.0260$ cm$ ^{3}$), and large (*n* = 180; ${\gt} 142.0260$ cm$ ^{3}$); FDG lesions were stratified as small (*n* = 167; ${\unicode{x2A7D} }45.7125$ cm$ ^{3}$), medium (*n* = 167; ${\gt} 45.7125$ and ${\unicode{x2A7D}} 191.6581$ cm$ ^{3}$), and large (*n* = 167; ${\gt} 191.6581$ cm$ ^{3}$). Statistical significance was assessed using paired Wilcoxon signed-rank tests with Holm correction for multiple comparisons. Asterisk annotations (*, **, ***) indicate the level of significance for comparisons between the CT model and all preceding models (* *p* <0.05, ** *p* <0.01, *** *p* <0.001). Letter annotations denote statistically significant differences (*p* <0.05) between successive model configurations: ‘*a*’ indicates the comparison between the from-scratch model and the natural-image model, ‘*b*’ between the natural-image and x-ray model, ‘*c*’ between the x-ray and CT-0.4 M model, and ‘*d*’ between the CT-0.4 M model and PET model. The placement of each letter indicates the higher-performing model within the corresponding comparison.

Model	Pretraining data size	Volume	PSMA Dice	FDG Dice
From-scratch	none	Small	0.323 (0.269)$ ^{\scriptsize*}$	0.500 (0.262)$ ^{\scriptsize***}$
		Medium	0.581 (0.269)$ ^{\scriptsize***}$	0.689 (0.203)$ ^{\scriptsize***a}$
		Large	0.713 (0.211)$ ^{\scriptsize***}$	0.705 (0.182)$ ^{\scriptsize***a}$

Natural-image	1689 M	Small	0.291 (0.273)$ ^{\scriptsize***}$	0.484 (0.295)$ ^{\scriptsize***}$
		Medium	0.576 (0.257)$ ^{\scriptsize***}$	0.602 (0.263)$ ^{\scriptsize***}$
		Large	0.693 (0.219)$ ^{\scriptsize***}$	0.669 (0.207)$ ^{\scriptsize***}$

x-ray	0.4 M	Small	0.325 (0.272)$ ^{\scriptsize***}$	0.578 (0.293)$ ^{\scriptsize***b}$
		Medium	0.599 (0.234)$ ^{\scriptsize***}$	0.723 (0.184)$ ^{\scriptsize***b}$
		Large	0.706 (0.196)$ ^{\scriptsize***b}$	0.760 (0.152)$ ^{\scriptsize***bc}$

CT	0.4 M	Small	0.350 (0.245)$ ^{\scriptsize***cd}$	0.599 (0.283)$ ^{\scriptsize***cd}$
		Medium	0.637 (0.201)$ ^{\scriptsize***cd}$	0.704 (0.213)$ ^{\scriptsize***d}$
		Large	0.767 (0.100)$ ^{\scriptsize***cd}$	0.746 (0.148)$ ^{\scriptsize***d}$

PET	0.01 M	Small	0.179 (0.226)$ ^{\scriptsize***}$	0.239 (0.241)$ ^{\scriptsize***}$
		Medium	0.413 (0.250)$ ^{\scriptsize***}$	0.361 (0.257)$ ^{\scriptsize***}$
		Large	0.595 (0.216)$ ^{\scriptsize***}$	0.422 (0.223)$ ^{\scriptsize***}$

CT	3 M	Small	**0.386 (0.305)**	**0.636 (0.292)**
		Medium	**0.655 (0.227)**	**0.780 (0.139)**
		Large	**0.741 (0.178)**	**0.825 (0.123)**

## 5. Conclusion

This study systematically evaluated the impact of self-supervised pretraining domain on PET/CT tumor segmentation using DINOv3 encoders within a standardized nnU-Net framework (table 1). Our results demonstrate that downstream performance is jointly influenced by both domain proximity and pretraining dataset scale (tables 2 and 3). First, at comparable dataset scale, CT-0.4 M outperformed x-ray-0.4 M for most evaluated endpoints, supporting the value of CT domain proximity for PET/CT segmentation. PET pretraining performed well for HECKTOR GTVp when 0.3 M PET samples were available. In autoPET, PET-0.01 M produced Dice values of 0.394 for PSMA and 0.339 for FDG, outperforming CT-0.01 M for PSMA but remaining below the larger CT-0.4 M and CT-3 M models for both autoPET targets. Second, comparing CT scales showed that larger and more diverse CT pretraining can further improve selected targets, particularly HECKTOR GTVn and autoPET FDG, although this benefit was not uniform for PSMA. In contrast, pretraining on low-proximity natural images did not improve over training from scratch despite its very large dataset size. In summary, CT-based post-training offers a favorable balance between domain relevance, data availability, and anatomical diversity for the PET/CT segmentation tasks evaluated here. Future work may explore larger PET-specific pretraining datasets to more fully separate modality alignment from data scale.

## Data Availability

No new data were created or analysed in this study. Supplementary data 1 available at https://doi.org/10.1088/1361-6560/aeb29f/data1.
